# Effect of Interval between Neoadjuvant Chemoradiotherapy and Surgery on Oncological Outcome for Rectal Cancer: A Systematic Review and Meta-Analysis

**DOI:** 10.1155/2016/6756859

**Published:** 2016-03-30

**Authors:** Xiao-Jie Wang, Zheng-Rong Zheng, Pan Chi, Hui-Ming Lin, Xing-Rong Lu, Ying Huang

**Affiliations:** ^1^Department of Colorectal Surgery, Union Hospital, Fujian Medical University, Fuzhou, Fujian 350001, China; ^2^Oncology Department, The Second Affiliated Hospital of Fujian Medical University, Quanzhou, Fujian 360000, China

## Abstract

*Aim*. To evaluate the influence of interval between neoadjuvant chemoradiotherapy (NCRT) and surgery on oncological outcome.* Methods*. A systematic search was conducted in PubMed, the Cochrane Library, and Embase databases for publications reporting oncological outcomes of patients following rectal cancer surgery performed at different NCRT-surgery intervals. Relative risk (RR) of pathological complete response (pCR) among different intervals was pooled.* Results*. Fifteen retrospective cohort studies representing 4431 patients met the inclusion criteria. There was a significantly increased rate of pCR in patients treated with surgery followed 7 or 8 weeks later (RR, 1.45; 95% CI, 1.18–1.78; and *P* < 0.01 and RR, 1.49; 95% CI, 1.15–1.92; and *P* = 0.002, resp.). There is no consistent evidence of improved local control or overall survival with longer or shorter intervals.* Conclusion*. Performing surgery 7-8 weeks after the end of NCRT results in the highest chance of achieving pCR. For candidates of abdominoperineal resection before NCRT, these data support implementation of prolonging the interval after NCRT to optimize the chances of pCR and perhaps add to the possibility of ultimate organ preservation.

## 1. Introduction 

The current standard of treatment for locally advanced rectal cancer, that is, stage cT3-4/Nx or cTx/N1-2 disease, consists of neoadjuvant chemoradiotherapy (NCRT), followed by radical total mesorectal excision (TME) (plus or minus further adjuvant chemotherapy) [1]. A recent published meta-analysis of randomized controlled trials suggested that preoperative chemoradiotherapy improves local control compared with surgery alone or surgery with neoadjuvant radiotherapy [2]. In addition, neoadjuvant therapy may result in complete eradication of all viable tumor cells from the primary tumor site as well as from the regional lymph nodes, representing pathological complete response (pCR) in this setting. Furthermore, complete response to preoperative NCRT is indicative of better long-term outcomes with low rates of local recurrence and distant failure [3].

A few small studies have recently investigated the influence of the length of the interval between NCRT and surgery (NCRT-surgery interval) on morbidity, resectability, and tumor response [4, 5]. However, the optimal NCRT-surgery interval that allows for maximal tumor regression is still unknown, and quantitative summarization of the supporting evidence of each time interval is not available. The purpose of this systematic review was, therefore, to examine the impact of the NCRT-surgery interval on the oncological outcome.

## 2. Material and Methods 

### 2.1. Data Sources and Searches

We searched PubMed, the Cochrane Library, and Embase databases for relevant articles published until February 1, 2015; no lower date limit was applied. The search was limited initially to English publications. Electronic database searches were performed with the Boolean combination [(Interval OR time OR timing) AND rectal AND (carcinoma OR cancer) AND (Chemoradiotherapy OR Radiotherapy OR radiation OR neoadjuvant)] in all fields. In addition, the reference lists of included studies and related publications were screened for additional trials.

### 2.2. Study Selection

Two authors independently screened each unique record identified by the searches. Clinical trials meeting the following criteria were included in the meta-analysis: (i) the study directly compared pCR rates between patients in whom surgery for rectal cancer was performed at different intervals after NCRT. (ii) Patients were reported to undergo neoadjuvant radiation-based therapy and concurrent chemotherapy before surgery. (iii) The pCR rates were reported according to longer interval (NCRT-surgery interval longer than cutoff point) and shorter interval (NCRT-surgery interval shorter than cutoff point) categories. Studies that defined more than 2 time intervals were also accepted. (iv) The study had to report comparable data on pCR rates after NCRT of each time interval category. We excluded studies that were not published as full reports, such as conference abstracts and letters to editors. In case of multiple publications on the same study, the most recent information was used. Discrepancies in study selection were resolved by consensus.

### 2.3. Data Extraction and Study Assessment

To avoid bias in the data-abstraction process, two authors assessed the quality, extracted the data, and assessed the risk of bias independently. Any discrepancies between the authors were resolved by consensus. Details about the author, publication year, number of patients, treatment information, patient baseline characteristics, definitions of the pCR, pCR rates, and sphincter preservation rates of different groups and prognosis results were extracted from the included studies. When studies compared pCR rates between more than 2 time intervals, data were collected from each group separately. Methodological quality was assessed using Newcastle-Ottawa quality assessment scale.

### 2.4. Statistical Analysis

Rates of pCR was the primary endpoint, and local recurrence rates, distant metastases rates, overall survival rates, and sphincter preservation rates were the secondary endpoints. All statistical analysis was implemented with Review Manager version 5.3 software (Cochrane Collaboration, Denmark). To calculate relative risk (RR), patients of longer interval group were compared only with those of shorter interval in the same clinical trial. RR together with the 95% confidence interval (CI) was used as summary statistics for dichotomous data. We explored a relationship between pCR rates and time intervals by dividing patients into six categories based on NCRT-surgery intervals. We assessed statistical heterogeneity with *I* ^2^ statistics. Statistical heterogeneity between groups was considered relevant for comparisons with *I* ^2^ statistics of >50%. The *Z*-test for overall effect and its two-sided *P* value were assessed. Significant difference was considered to be present for *P* < 0.05.

### 2.5. Assessment of Bias Risk of the Included Studies and Sensitivity Analysis

Publication bias was not evaluated because of the small number of studies included in each subgroup. Sensitivity analysis was performed for studies with same definition of pCR.

## 3. Results

**Figure 1:**
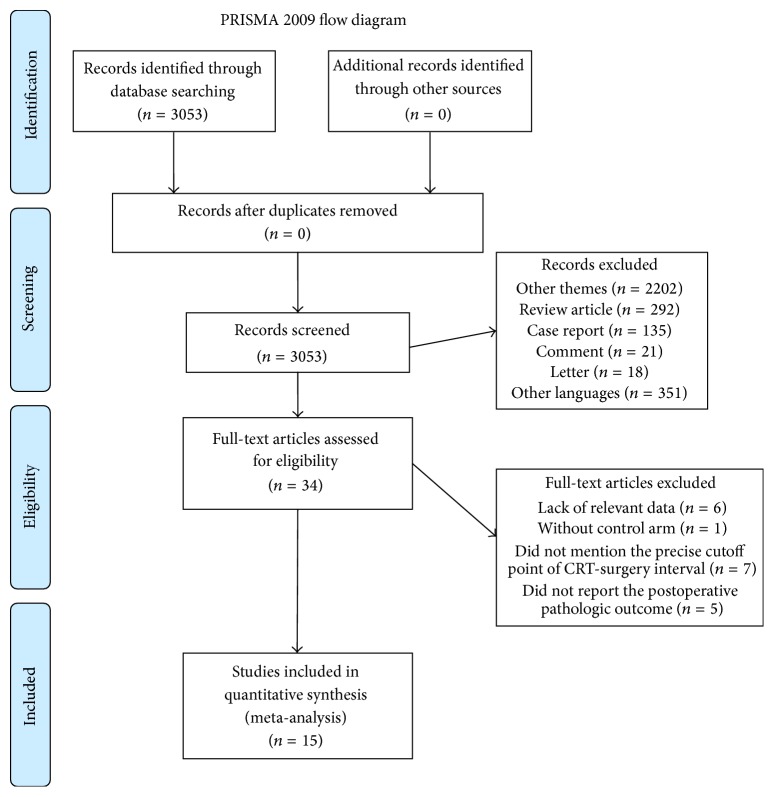
Flowchart of search process.

Our search yielded a total of 3053 potentially relevant clinical studies, of which 2202 were among other themes. We then excluded review articles (*n* = 292), case reports (*n* = 135), comments (*n* = 21), letters (*n* = 18), and articles written in other languages (*n* = 351). In total, 34 articles were retrieved for full-text review. Of these, 6 articles were excluded due to lack of relevant data, 1 due to lack of a control arm, 7 due to failure to define a precise cutoff point of NCRT-surgery interval, and 5 due to failure to report postoperative pathologic outcome. The remaining 15 studies comprised a total of 4431 individuals, which constituted the material for the current review [6–20] (see Figure 1 for the selection process of these studies).

### 3.1. Characteristics of Studies Included in the Meta-Analysis

Fifteen retrospective cohort studies were included [6–20]. The studies included in this review are heterogeneous in the time intervals that they compared, ranging from ≤5 weeks versus >5 weeks to ≤12 weeks versus >12 weeks. All of the studies reported on patients who received NCRT. Although the exact nature of preoperative radiotherapy differed between the studies examined in this review, the majority of studies administered 45 to 50.4 Gy delivered over a period of 5 to 6 weeks. All of the studies reported on patients who received 5-fluorouracil- (FU-) based chemotherapy, though the chemotherapy regimens used varied. The common factors influencing NCRT-surgery intervals reported by most studies were surgeons' policy regarding the timing of operation, bed availability on the surgical wards, and comorbidities. The precise definition of complete pathological response was consistent among included studies, except Fang et al. [16] who defined pCR as “pT0 and any pN,” and one patient who had a lymph node metastasis (ypT0N2) was therefore included in pCR group. Tulchinsky et al. [9] pooled pCR rates and near-pCR rates together for the analyses. The average methodological quality score was 6.7 (minimum 5, maximum 7) (Table 1).

The clinicopathologic data of the study populations are summarized in Table 2. No differences in average height of tumors from the anal verge were identified between shorter and longer intervals in any of the studies, except Sirohi et al. [20] who reported that height of tumors from the anal verge in shorter interval group was higher (6 cm versus 4 cm; *P* = 0.045). The majority of studies reported that neoadjuvant therapy was given to all patients with stage II or stage III rectal cancer, or with tumors that threatened circumferential resection margin. None of the included studies reported a significant difference in distribution of clinical T-stage and N-stage between shorter and longer intervals cohorts.

### 3.2. Complete Pathological Response

Four studies reported a significantly increased rate of pCR after neoadjuvant therapy when patients were operated on at an interval >7 weeks [9, 13, 18, 19], and de Campos-Lobato et al. [11] found that a waiting interval of ≥8 weeks was associated with a higher rate of pCR. The highest pCR rates were observed in patients undergoing surgery on 10 to 11 weeks after the end of NCRT in the large series reported by Sloothaak et al. [14]. Seven studies reported an insignificant trend toward increased pCR rates in longer interval group [6–8, 12, 15, 17, 20]. No increase in rates of complete response was identified in 2 studies [10, 16].

### 3.3. Meta-Analysis of pCR Rate

**Figure 2:**
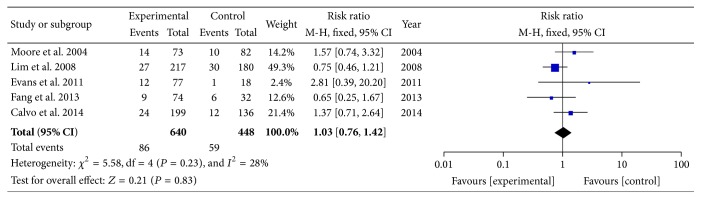
Standard forest plot of the RR for pCR rates comparing longer time intervals with shorter intervals at 6 weeks.

**Figure 3 fig3:**
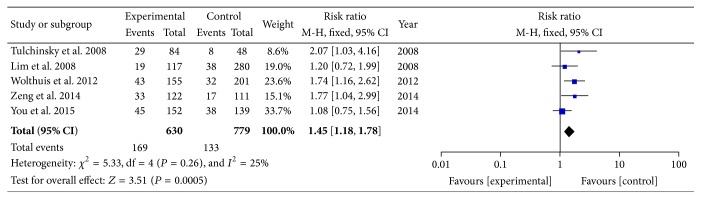
Standard forest plot of the RR for pCR rates comparing longer time intervals with shorter intervals at 7 weeks.

**Figure 4 fig4:**
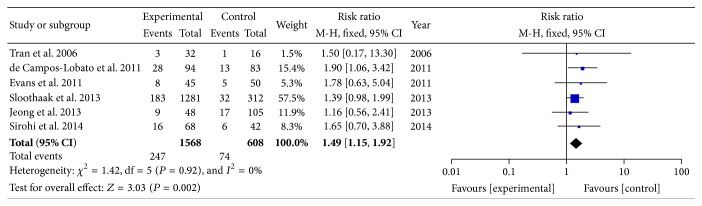
Standard forest plot of the RR for pCR rates comparing longer time intervals with shorter intervals at 8 weeks.

**Figure 5:**
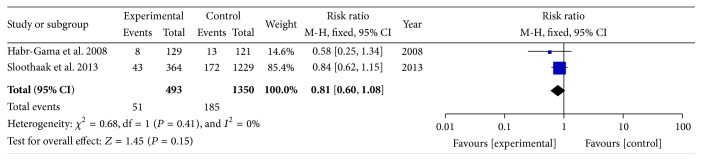
Standard forest plot of the RR for pCR rates comparing longer time intervals with shorter intervals at 12 weeks.

The reported pCR rates ranged from 8.3% to 28.0% [6–20]. In order to determine the particular contribution of time intervals of NCRT-surgery to the occurrence of pCR, a meta-analysis was performed to calculate the RR associated with longer time intervals at beyond 5, 6, 7, 8, 10, or 12 weeks when compared to shorter intervals (Table 3, Figures 2 [Fig fig3] [Fig fig4]–5). No heterogeneity was found among these studies included in the analysis (*I* ^2^ < 50% each). Using a fixed-effect model, there was a significantly increased rate of pCR in patients treated with surgery followed 7 or 8 weeks later (RR, 1.45; 95% CI, 1.18–1.78; and *P* < 0.01 and RR, 1.49; 95% CI, 1.15–1.92; and *P* = 0.002). No significant differences were found between shorter and longer intervals cohorts with respect to rate of pCR in earlier cutoff points of 5 and 6 weeks (RR, 0.67; 95% CI, 0.40–1.12; and *P* = 0.13 and RR, 1.03; 95% CI, 0.76–1.42; and *P* = 0.83), and further extension beyond 10 or 12 weeks did not offer further advantages in increasing pCR rates (RR, 0.83; 95% CI, 0.65–1.06; and *P* = 0.13 and RR, 0.81; 95% CI, 0.60–1.08; and *P* = 0.15). Sensitivity analyses excluding data from Tulchinsky et al. [9] to control for patient with near-pCR did not alter the results substantially (RR, 1.39; 95% CI, 1.12 to 1.73; and *P* = 0.003). Data from Fang et al. [16] was also excluded for sensitivity analyses because of different definition of pCR, and result showed no change (RR, 1.09; 95% CI, 0.78 to 1.52; and *P* = 0.61).

**Figure 6:**
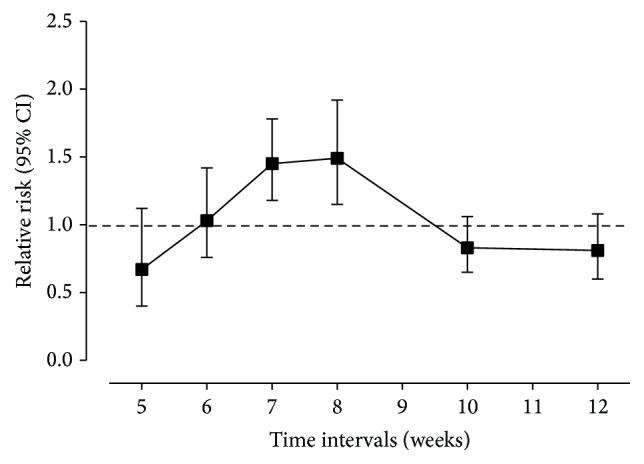
The relationship between time intervals and RR of pCR rates. The center of each black square is placed at the summary point estimate, and each vertical line shows the 95% confidence interval about the summary estimate.

We then plotted the pooled RR and 95% CI of pCR rates of different intervals in a line chart. The highest summary point estimate in the RR of pCR rates was observed in patients receiving surgery beyond 8 weeks after the end of NCRT, which was associated with an approximately 49% higher chance of achieving pCR than patients who were operated on less than 8 weeks after the end of NCRT. The corresponding figure for ≥7 weeks' cutoff point was 45% (Figure 6).

### 3.4. Sphincter Preservation

Thirteen of the included studies reported rate of sphincter preservation (Table 4). None of the included studies reported a significant increase in rates of sphincter preservation with a longer interval. Habr-Gama et al. [10] reported a lower rate of sphincter preservation among patients undergoing surgery after a longer interval of >12 weeks, as patients in this study with a suspected cCR after NCRT were enrolled in a watch-and-wait protocol and were not managed by surgery until recurrence occurred.

### 3.5. Long-Term Outcome

Twelve of the included studies reported long-term outcomes (Table 4). Six of these found no significant difference in local recurrence rates, or local control rate, and distant metastases rate between patients who were operated on after shorter or longer intervals. A significantly higher rate of local recurrence was reported in patients undergoing surgery after a shorter interval (<7, 8 weeks) reported by de Campos-Lobato et al. [11] and Zeng et al. [18] (10.5% versus 1.2%, *P* = 0.04, and 12.9% versus 4.8%, *P* = 0.025, resp.). Tulchinsky et al. [9] reported a significant increase in distant metastases rate when surgery was performed after a shorter interval <7 weeks (19% versus 6%, *P* = 0.02). A significantly higher 5-year free-from-recurrence rate was reported in patients undergoing surgery after an interval of >8 weeks reported by Wolthuis et al. [13] (73% versus 83%, *P* = 0.026). In terms of overall survival, six studies found no significant difference between patients who were operated on after shorter or longer intervals except 1 study, in which Calvo et al. [17] identified a significant association between increased 5-year overall survival with prolonged interval of ≥6 weeks.

## 4. Discussion

The optimal timing for surgery after neoadjuvant treatment for rectal cancer remains at large. The justification for an effort to examine optimal timing of surgery after preoperative radiation therapy stems from the Lyon R90-01 study [21], the only randomized trial to date that examines the time interval to surgery, in which outcomes after short (less than 2 weeks) and long (6–8 weeks) intervals following preoperative radiotherapy were compared. The longer interval was correlated with a significantly higher proportion of pathologic downstaging. Furthermore, this trial was the only one that demonstrated an increase of sphincter preservation following longer interval to surgery. Therefore, the 6–8-week interval between NCRT and surgery has become routine practice for rectal cancer. However, patients in this study received a currently unusual radiation dose (39 Gy in 13 fractions), did not routinely undergo TME, and did not receive preoperative chemotherapy. Furthermore, higher safe dose of radiation therapy (3 Gy per fraction) might impact the rate of sphincter preservation. Another ongoing multicentric randomized controlled trial (the GRECCAR6 study) only compares 7 and 11 weeks of delay between the end of NRCT and surgery of rectal cancer [22]. There is no previous studies evaluated the association between each time interval and rate of pCR.

Tumor regression and radiation-induced necrosis are a time-dependent phenomenon [23]. The effects of chemoradiotherapy are based on the cell cycle, and oftentimes multiple cycles administered over the course of several months are necessary before effects are seen [24]. Therefore, there is enthusiasm for prolonging the currently accepted interval of 6–8 weeks in order to maximize the downstaging effect of NCRT and subsequently increase the pCR rate [25]. But will a longer interval even result in a superior rate of pCR? Dolinsky et al. [26] reported the rates of primary tumor downstaging were 42%, 58%, and 71% for patients with intervals of <6, 6–8, and >8 weeks, respectively, but an increase in the time interval did not affect the likelihood of achieving a pCR (OR 0.97, 95% CI, 0.78–1.21, and *P* = 0.8) in multivariate analysis. For short-course radiotherapy, there are 4 phase-III studies in the literature with randomized intervals. Two of these studies compared short-course radiotherapy and immediate surgery with short-course radiotherapy and delayed surgery and found that the ypCR rate was about 10% higher in the delayed-surgery group [27, 28]. The comparison of short-course radiotherapy and delayed surgery with long-course chemoradiation in another randomized study revealed that the pCR rate was higher in the long-course chemoradiation groups [29]. An interim analysis of a multicentre randomized study that compared short-course radiotherapy and consolidation chemotherapy with long-course chemoradiation revealed a higher pCR rate in the short-course radiotherapy group [30].

A published meta-analysis demonstrated that a longer waiting interval (more than 6–8 weeks) from the end of NCRT increases the rate of pCR by 6% (RR = 1.42, 95% CI, 1.19–1.68, and *P* < 0.0001), with similar long-term outcomes and complication rates [31]. However, they failed to perform subgroup analysis according to each particular time point. As there was lack of consistency in the time intervals examined by the included studies, heterogeneity may well exist when data of all time intervals were pooled together. After including more trials (including 4 new published trials with 969 patients), we pooled data from different time intervals to perform a subgroup analysis. The present data demonstrated that delaying surgery until the seventh or eighth week after NCRT significantly increased rate of pCR in patients with rectal cancer (increasing rate by 49% and 45%, resp.). Notably, no significant improvement of pCR rate was found when patients had operation beyond commonly accepted 6 weeks, as 6-week point is probably insufficient to reveal relevant differences. Thus, we establish the optimal window between 7-8 weeks to intervene surgically within the established 6–8 weeks' window.

There is, however, less data published regarding the results of increasing the interval prior to surgical intervention (>10 weeks). Our data suggests that no significant difference in pCR rate was found when patients had a waiting interval of ≥10 or 12 weeks. However, a potential limitation in our meta-analysis is that only two studies were included in these longer interval subgroups. Garcia-Aguilar et al. [23] indicated that patients operated on 11~13 weeks after NCRT had a pCR rate of 25%, compared to 18% for patients operated on 6~8 weeks after NCRT (*P* = 0.022). However, Stein et al. [24] did not find increased rate of pCR when surgery was performed at 10 to 14 weeks (14%) in comparison with surgery at 4 to 8 weeks (21%) after NCRT. In a recent published exploratory phase-2 trial, Garcia-Aguilar et al. [32] showed that adding up to six cycles of mFOLFOX6 chemotherapy between NCRT and surgery, meanwhile, delaying surgery after NCRT, increased the pCR rate. The increased number of cycles was significantly associated with an increased proportion of patients who achieved a pCR. The patients assigned to receive six cycles of mFOLFOX6 after chemoradiation with surgery 19 weeks after NCRT achieved a highest rate of pCR of 38%, which is one of the highest proportions reported so far for stages II-III rectal cancer. However, the excellent clinical response was probably not due to longer interval but preoperative chemotherapy. In addition, a few issues need to be addressed. Waiting longer clearly benefits those patients that achieve a pCR, but over 20% do not respond to preoperative therapy and, in fact, the primary tumor continues to grow [33]. Another concern of delaying surgery is that longer intervals after preoperative radiation may increase the risk of emergence of distant subclinical tumor, which can grow to a metastasis-yielding volume and lead to the development of distant metastases [34]. Despite several studies reporting promising use of imaging technology to help in monitoring disease response during preoperative treatment, no robust imaging technology has been established for widespread clinical use [35, 36]. Based on these concerns, further delaying time interval (>10 weeks) may be inappropriate.

Tumor response to NCRT has been shown to be a predictor of less propensity for local or distant recurrence and improved survival [16]. Indications of tumor regression include downstaging, downsizing, complete, or nearly complete response. As the definitions of downstaging and downsizing varied widely among studies [37], complete pathological response to neoadjuvant therapy has become a widely measured endpoint in rectal cancer clinical trials [38]. In this study, we chose to define pCR as absence of viable adenocarcinoma cells in the surgical specimen, including primary tumor and lymph nodes. There is marked heterogeneity in reported pCR rates across included studies (8.3%–28.0%), probably due to different neoadjuvant therapy protocols and patients with different stages enrolled in. The sensitivity analyses were performed excluding the study in which patients with pCR (*n* = 26) and near-pCR (*n* = 11) were included [9], and another study in which one patient with pT0N2 stage was included in pCR group [16]. Results of sensitivity analyses suggest our findings were robust.

NCRT was reported to achieve tumor downsizing and downstaging, which might cause an increase in the tumor distance from the anal verge, meanwhile increasing the likelihood of sphincter preservation [39]. However, none of the studies included in this review reported a significant increase in rates of sphincter preservation with a longer interval. Although the longer interval to surgery was associated with a higher pCR rates in 4 phase-III trials regarding short-course radiotherapy, the sphincter preservation rate and long-term outcome were similar [27–29, 32]. When comparing with postoperative chemoradiotherapy, the benefit of sphincter preservation and survival was not confirmed for preoperative chemoradiotherapy by results of the German CAO/ARO/AIO-94 randomized phase-III trial [40, 41]. In the phase-II trial by Garcia-Aguilar et al., waiting longer than 19 weeks was not associated with an increase in the proportion of patients who had a sphincter-saving procedure. However, patients with good response who refused to undergo TME but local excision were excluded from analyses [32]. Though longer interval was not demonstrated to increase in conventional sphincter-saving procedure (e.g., anterior resection), it can increase the rate of pCR, meanwhile, increasing the proportion of organ preservation (e.g., local excision [42] or watch-and-wait policy [43, 44]) for patients with clinical complete response.

Whether there is an association between time interval and prognosis remains controversial. The meta-analysis performed by Petrelli et al. reported similar long-term outcomes between longer and shorter interval groups, but this meta-analysis redichotomised all of the patients into two groups according to the 6–8 weeks' interval [31]. In the current review, local control, distant metastasis, and overall survival were reported to be similar between longer and shorter interval in most of included studies, though a few studies reported an association between longer intervals with better long-term outcome. We failed to perform a meta-analysis of survival data of each time interval group to compare prognosis of all of the possible intervals, as data were not available. Further studies investigating this are warranted.

Our study has the following limitations. First, all data were extracted from retrospective studies, but the majority of included studies reported patients with similar baseline demographic and oncologic characteristics in both groups. Tulchinsky et al. [9] reported that patients operated on at an interval >7 weeks were older at operation (*P* = 0.007), but they were able to show that age at operation was not a predictor for pCR and near-pCR (*P* = 0.57). Moreover, while most analyses were conducted from a retrospective perspective, the data used were collected prospectively in the highly standardized manner; neoadjuvant therapy schedule and our primary outcome of interest, pCR, are all routinely collected and objectively measured, thereby minimizing the problems of missing data and reporting bias. Second, the time intervals after NCRT showed significant heterogeneity among the included studies. As surgeons' policy regarding the timing of operation was reported to be the most common factor influencing NCRT-surgery intervals by most studies, various potential confounders could have been involved in the choice of interval times for the cases considered. For instance, patients with progressive or stable disease after NCRT might have surgery without further delay after completing NCRT, which caused selection bias. Third, we failed to perform a meta-analysis of survival data of each time interval group. Although time intervals of 7-8 weeks significantly increase chances of achieving pCR in present study, it is unclear whether this translates into long-term clinical benefit.

## 5. Conclusion

The results of this review demonstrate that performing surgery 7-8 weeks after the end of NCRT significantly increases rates of pCR. Increasing the interval prior to surgical intervention alone has no impact on long-term survival. For candidates of abdominoperineal resection before NCRT, these data support implementation of prolonging interval after NCRT to optimize the chances of pCR and perhaps add to the possibility of ultimate organ preservation. This is best addressed in the context of a randomized control trial.

## Figures and Tables

**Table 1:** Main characteristics of the studies included in the meta-analysis.

Trial	Year	Study period	Interval	Radiotherapy schedule	Chemotherapy schedule	Delay reason	pCR definition	Quality score (out of 9)
Moore et al. [6]	2004	1980–2002	44 days	50.4 Gy, 46.8 Gy in twenty-six 1.8 Gy fractions	5-FU/LV or irinotecan	NA	Tumors that were ypT0N0, or only acellular pools of residual mucin were noted	7

Tran et al. [7]	2006	1997–2004	8 weeks	45–50.4 Gy	5-FU	According to the surgeons' preference and because it was typically influenced by tumor size/bulk and the perceived need for tumor shrinkage for resectability and/or sphincter salvage	NA	7

Lim et al. [8]	2008	2002–2006	5, 6, and 7 weeks	50.4 Gy, 45 Gy in twenty-five 1.8 Gy fractions over 5.5 weeks	5-FU/LV or capecitabine or irinotecan/capecitabine	According to the surgeons' preference and their policy regarding the timing of operation	NA	7

Tulchinsky et al. [9]	2008	2000–2006	7 weeks	45–50.4 Gy over 5.5 weeks	5-FU	According to bed availability on the surgical ward	pCR and near-pCR rates (the latter being defined by the finding of microscopic foci of adenocarcinoma in the rectal wall with no cancer cells in the lymph nodes)	6

Habr-Gama et al. [10]	2008	1991–2005	12 weeks	50.4 Gy over 6 weeks	5-FU/LV	According to the medical conditions as infections and acute myocardial ischemia, among others; hospital bed and operating room availability; and suspected cCR	ypT0N0M0	7

de Campos-Lobato et al. [11]	2011	1997–2007	8 weeks	50.4 Gy	5-FU	Attributed to logistical, scheduling, and clinical factors	Absence of viable adenocarcinoma cells in the surgical specimen, including primary tumor and lymph nodes	7

Evans et al. [12]	2011	2005–2008	6, 8 weeks	45 to 54 Gy, 1.8 Gy per fraction over 5 to 6 weeks	5-FU/oxaliplatin or 5-FU/irinotecan or 5FU plus other^a^	Attributed to scheduling and comorbidities	NA	6

Wolthuis et al. [13]	2012	2000–2009	7 weeks	45 Gy in twenty-five 1.8 Gy fractions	5-FU	Attributed to logistical factors, hospital bed availability, and surgeons' and patients' scheduling preferences	Mucous lakes without identifiable carcinoma cells	7

Sloothaak et al. [14]	2013	2009–2011	8, 10, and 12 weeks	50 Gy in twenty-five 2.0 Gy fractions/20.4 Gy in twenty-eight 1.8 Gy fractions/45 Gy in twenty-five 1.8 Gy fractions	5-FU ± oxaliplatin	NA	ypT0N0M0	7

Jeong et al. [15]	2013	2008-2009	8 weeks	50.4 Gy, 45 Gy in twenty-five 1.8 Gy fractions over 5 weeks	5-FU/LV, CPT-11/S-1 (16%), TS-1/irinotecan (12%), or Xeloda (6%)	Attributed to logistics, scheduling, and other clinical factors	NA	7

Fang et al. [16]	2013	2004–2010	6 weeks	50.4 Gy in twenty-eight fractions over 5.5 weeks	5-FU	NA	T0 any N	5

Calvo et al. [17]	2014	1995–2012	6 weeks	50.4 Gy, 45 Gy in twenty-five 1.8 Gy fractions over 5 weeks	5-FU + tegafur/Folfox-4	Attributed to logistics, scheduling, surgeon discretion, and other clinical factors	A complete absence of tumor cells in the resected specimen (ypt0) and the resected nodes (ypn0)	6

Zeng et al. [18]	2014	2005–2012	7 weeks	50.0 Gy, 2.0 Gy per fraction	Capecitabine	Attributed to logistical factors, such as hospital bed availability, surgeons' and patients' scheduling preferences	ypT0N0	7

You et al. [19]	2015	2004–2012	7 weeks	50.0 Gy, 46 Gy in twenty-three 2.0 Gy fractions, and additional 4 Gy injected into the primary tumour	Folfox-6 or Xelox	NA	No cancer cells in either the primary tumour samples or the retrieved lymph nodes, or mucous lakes without identifiable carcinoma cells	7

Sirohi et al. [20]	2014	2012-2013	60 days	50.0 Gy, 2.0 Gy per fraction	Capecitabine	Attributed to a long waiting list for surgery or patients' scheduling preferences	NA	7

^a^Other: cetuximab or bevacizumab.

NA, data not available; 5-FU, 5-fluorouracil.

**Table 2:** The clinicopathologic data of the study populations.

Trial	Interval	Patient number	Mean age (years)	Gender		Interval range	Height from anal verge, cm	Preneoadjuvant therapy stage				pCR%	pCR (*n*)	*P* value
				Male	Female			I	II	III	IV			
Moore et al. [6]	≤44 days	82	59	52	30	15∼206 days	6.1	3	28	51	0	12	10	0.19
	>44 days	73	62	46	27		6.4	3	21	49	0	19	14	

Tran et al. [7]	≤8 weeks	16	62.3	10	6	8∼43 weeks	≤10 (NS)	0	8	8	0	6	1	0.19
	>8 weeks	32	58.1	18	14			1	18	11	1	9	3	

Lim et al. [8]	2∼41 days	217	55.3	138	79	35.6 ± 3.3 days	4.8 ± 2.1		43 (I + II)	174	0	13.8	30	0.74
	42∼56 days	180	57.5	125	55	50.1 ± 4.4 days	4.8 ± 2.1		39 (I + II)	141	0	15	27	

Tulchinsky et al. [9]	≤7 weeks	48	59	35	13	13∼173 days	5.6	0	39	6	0	16.7	8	0.03
	>7 weeks	84	64	54	30		6.2	0	63	17	0	34.5	29	

Habr-Gama et al. [10]	≤12 weeks	121	56.9	65	56	9.8 ± 2.2 weeks	4.1 ± 1.7	10	50	23	0	13	11	0.2
	>12 weeks	129	60	83	46	25.8 ± 12.3 weeks	3.9 ± 1.8	7	57	25	0	8	6	

de Campos-Lobato et al. [11]	<8 weeks	91	57	129	48	4∼14 weeks	5.5 (4–7)	0	53	28	0	16.2	13	0.027
	≥8 weeks	86					6 (3–7)	0	50	39	0	31.1	28	

Evans et al. [12]	<6 weeks	18	66	60	35	3 days∼24 weeks	NS	NA^a^	0	27	NA^a^	5.6	1	NA
	6∼8 weeks	32						NA^a^	6	23	NA^a^	12.5	4	
	>8 weeks	45						NA^a^	10	34	NA^a^	17.8	8	

Wolthuis et al. [13]	≤7 weeks	201	64	124	77	28∼103 days	≤10	0	27	174	0	15.9	32	0.006
	>7 weeks	155	62	109	46	50∼103 days		0	14	141	0	28.4	43	

Sloothaak et al. [14]	<8 weeks	312	63	200	112	1∼80 weeks	NS		74 (I + II)	198 (III + IV)		10.3	32	0.013
	8∼9 weeks	511	63	327	184				105 (I + II)	354 (III + IV)		13.1	67	
	10∼11 weeks	406	64	244	162				68 (I + II)	306 (III + IV)		18	73	
	≥12 weeks	364	64	229	135				68 (I + II)	255 (III + IV)		11.8	43	

Jeong et al. [15]	<8 weeks	105	58.4	79	26	4∼14 weeks	4.8 ± 2.6	6	30	69	0	16.2	17	0.817
	≥8 weeks	48	56.4	38	10		4.7 ± 2.7	3	7	38	0	18	9	

Fang et al. [16]	5∼6 weeks	32	NA	NA	NA	NA	mid and low rectal cancer	0	56	50	0	18.8	6	0.372
	≥6 weeks	74										12.2	9	

Calvo et al. [17]	<6 weeks	136	65.5	91	45	4∼8 weeks	7.0		47^a^	84^a^		8.8	12	0.34
	≥6 weeks	199	66.0	109	90		7.0		45^a^	147^a^		12.1	24	

Zeng et al. [18]	≤7 weeks	111	59	62	49	25–105 days	NS		32	79		15.3	17	0.029
	>7 weeks	122	59	68	54				28	94		27.1	33	

You et al. [19]	≤7 weeks	139	55	96	43	4–14 weeks	NS		40	99		27.3	38	0.030
	>7 weeks	152	56	108	44				50	102		29.6	45	

Sirohi et al. [20]	≤60 days	42	50	28	14	6–474 days	6		NA			14	6	0.24
	>60 days	68	48	50	18		4 (*P* = 0.045)					24	16	

^a^with data missing.

NA: data not available.

**Table 3:** Meta-analysis of pCR rate according to time intervals.

Time intervals	Number of studies	Longer time intervals (*n*)/shorter intervals (*n*)	Test for heterogeneity		RR	95% CI	*P* value
			*I* ^2^	*P* value			
5 weeks	1	309/88	—	—	0.67	0.40–1.12	0.13
6 weeks	5	640/448	28%	0.23	1.03	0.76–1.42	0.83
7 weeks	5	630/779	25%	0.26	1.45	1.18–1.78	<0.01
8 weeks	6	1568/608	0%	0.92	1.49	1.15–1.92	0.002
10 weeks	1	770/543	—	—	0.83	0.65–1.06	0.13
12 weeks	2	493/1350	0%	0.41	0.81	0.60–1.08	0.15

CI, confidence interval.

**Table 4:** The sphincter-preserving procedure rate and long-term outcome.

Trial	Interval	Sphincter-preserving procedure (%)	*P* value	Median follow-up (months)	Local recurrence	Distant metastases	Overall survival
Moore et al. [6]	≤44 days	79.3	0.54		NA		
	>44 days	83.6					

Tran et al. [7]	≤8 weeks	75.0	1.0	27.7		31% versus 34% (*P* = 0.53)	
	>8 weeks	75.0					

Lim et al. [8]	2∼41 days	83.9	0.688	31	Local recurrence-free survival (*P* = 0.1165)		*P* = 0.8386
	42∼56 days	82.2					

Tulchinsky et al. [9]	≤7 weeks	62.5	0.95	35	6% versus 4% (*P* = 0.67)	19% versus 6% (*P* = 0.02)	
	>7 weeks	61.9		31			

Habr-Gama et al. [10]	≤12 weeks	52.9	0.003	46	34% versus 30% (*P* = 0.75)	12% versus 12% (*P* = 0.999)	
	>12 weeks	34.1					

de Campos-Lobato et al. [11]	<8 weeks	72.3	0.86	59	10.5% versus 1.2% (*P* = 0.04)	17.7% versus 14.1% (*P* = 0.85)	85.5% versus 88.2% (*P* = 0.74)
	≥8 weeks	72.3					

Evans et al. [12]	<6 weeks	83.3	NA		NA		
	6∼8 weeks	84.4					
	>8 weeks	73.3					

Wolthuis et al. [13]	≤7 weeks	86.1	0.18	4.99 years	5 years free from recurrence: 73% versus 83% (*P* = 0.026)		5-year cancer related survival: 83% versus 91% (*P* = 0.046)
	>7 weeks	78.7		4.73 years			

Sloothaak et al. [14]		NA	NA		NA		

Jeong et al. [15]	<8 weeks	82.4	0.341	38	3 years: 7.8% versus 12.7% (*P* = 0.279)	3 years: 24% versus 18.9% (*P* = 0.427)	3 years: 90.2% versus 87.2% (*P* = 0.825)
	≥8 weeks	75					

Fang et al. [16]	5∼6 weeks	NA	NA	32.2	3.1% versus 10.8% (*P* = 0.147)	31.3% versus 14.9% (*P* = 0.052)	
	≥6 weeks						

Calvo et al. [17]	<6 weeks ≥6 weeks	64 68.3	0.4	71	5-year local control: 90.4% versus 94.5% (*P* = 0.123)		5 years: 55.9 versus 70.4% (*P* = 0.014)


Zeng et al. [18]	≤7 weeks	59.5	0.335	42	3 years: 12.9% versus 4.8% (*P* = 0.025)	3 years: 14.4% versus 12.6% (*P* = 0.651)	3 years: 89.0% versus 94.5% (*P* = 0.651)
	>7 weeks	65.6					

You et al. [19]	≤7 weeks >7 weeks	55.4 57.2	0.832	55 49	5-year DFS: 74.7% versus 66.8% (*P* = 0.248)		5 years: 84.4% versus 75.3% (*P* = 0.679)


Sirohi et al. [20]	≤60 days	62	0.357	13	Overall recurrence: 14% versus 21% (*P* = 0.405)		
	>60 days	53					

NA = data not available.

## Abbreviations

NCRT: Neoadjuvant chemoradiotherapy
pCR: Pathological complete response
RR: Relative risk
CIs: Confidence intervals.
